# Recent Progress on Transition Metal Nitrides Nanoparticles as Heterogeneous Catalysts

**DOI:** 10.3390/nano9081111

**Published:** 2019-08-02

**Authors:** A.B. Dongil

**Affiliations:** 1 Institute of Catalysis and Petrochemistry, CSIC, c/Marie Curie No. 2, Cantoblanco, 28049 Madrid, Spain; a.dongil@csic.es; 2 UA UNED-ICP (CSIC) Group Des. Appl. Heter. Catal, 28049 Madrid, Spain

**Keywords:** heterogeneous catalysis, transition metal nitrides

## Abstract

This short review aims at providing an overview of the most recent literature regarding transition metal nitrides (TMN) applied in heterogeneous catalysis. These materials have received renewed attention in the last decade due to its potential to substitute noble metals mainly in biomass and energy transformations, the decomposition of ammonia being one of the most studied reactions. The reactions considered in this review are limited to thermal catalysis. However the potential of these materials spreads to other key applications as photo- and electrocatalysis in hydrogen and oxygen evolution reactions. Mono, binary and exceptionally ternary metal nitrides have been synthetized and evaluated as catalysts and, in some cases, promoters are added to the structure in an attempt to improve their catalytic performance. The objective of the latest research is finding new synthesis methods that allow to obtain smaller metal nanoparticles and increase the surface area to improve their activity, selectivity and stability under reaction conditions. After a brief introduction and description of the most employed synthetic methods, the review has been divided in the application of transition metal nitrides in the following reactions: hydrotreatment, oxidation and ammonia synthesis and decomposition.

## 1. Introduction

In the last decade strong interest has emerged in the field of nitrogen-doped catalysts, especially since new carbon nanostructures have been successfully synthetized. In those structures nitrogen can be easily inserted as heteroatoms due to its similarity to carbon, and as a doping agent it offers new possibilities in the field of catalysis [1]. Nitrogen confers basic sites for optimal reactant adsorption and an excellent electron density that may improve the catalytic performance due to the better dispersion of the active phase and the changes in the electronic properties [2].

Transition metal nitrides have been traditionally employed as catalysts in reactions such as hydrodesulfurization or ammonia synthesis [3]. Nowadays, researchers face new challenges in catalytic transformations to find active and selective catalysts in the fields of energy or biomass including reactions such as hydrodeoxygenation, water-gas shift and CO and CO_2_ hydrogenation among others [4]. 

Noble metals have been widely studied on those reactions. However economical catalysts based on abundant materials are more suitable for industrial applications. Hence, the need of searching new catalyst able to replace noble metals and the positive effect of nitrogen doping in several transformations, have brought up renewable attention to transition metal nitrides. These materials can donate electrons from the nitrogen atom and possesses high chemical, mechanical and thermal stability. Also importantly for thermal catalytic applications, when optimal synthesis conditions are employed, nitrides can reach relatively high surface areas of at least 90 m^2^/g [5].

**Figure 1:**
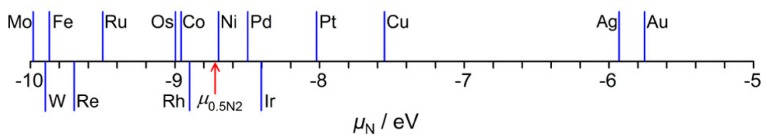
Value of the nitrogen chemical potential (μN) where the nitrogen covered surface becomes more stable than the clean metal surface; Reproduced from [18], with permission from American Chemical Society, 2018.

Transition metal nitrides are compounds in which nitrogen is incorporated into the interstitial sites of the metal structure. Since the size of the nitrogen atom is small (0.065 nm), it fulfills the Hägg rule (i.e., the ratio of the radii of non-metal to metal is less than 0.59, this allowing the formation of simple typical structures as compiled in Figure 1 [6]. 

As it can be expected the reactivity of the resulting transition metal nitride is different from that of the parent metal. In general, two main effects can be considered when nitrides are used in catalysis [7,8]:

(a) Ligand effect: Nitrides have a different electronic structure compared to the parent metal. This is due to the charge transfer from the metal to the non-metal, hybridization of the metal d-states with the non-metal sp-states, and expansion of the metal–metal lattice spacing. These changes in the electronic structure, modify the chemical reactivity of the parent metal so that the strenght of adsorption of reactants and products is similar to noble metals, this improving the selectivity of the reactions.
(b) Ensemble effect: When nitrogen is located on the metal surface, it may decrease the number of available metal sites, also creating different adorption sites. This effect can be somehow tuned by changing the metal to nitrogen ratio.

## 2. Synthesis and Structural Properties

Metal nitrides can be prepared among other methods by thermal treatment of the metal precursor obtained from template methods [9], ball milling [10,11] or temperature programmed reaction/reduction procedures [12]. 

Template methods are based on the use of a sacrificial template, normally MgO, to which a mixture containing the metal precursor is added followed by thermal treatment at high temperatures and extensive wash to remove the MgO template. In this way Metal-N entities are well dispersed in a carbon based matrix. In the ball milling method, the solid metal precursor either commercial or lab-made are submitted to milling under pressure and connected to a supply of the nitridation agent, N_2_ or NH_3_. 

So far, the most employed method to synthetize transition metal nitrides is a temperature programmed reaction/reduction procedure. The method consists in a reductive nitriding treatment under a gas flow (i.e., NH_3_ or a mixture of N_2_:H_2_) of the corresponding metal oxide precursor to produce the nitride [12]. The characteristics of the final nitride depend on the precursor and preparation conditions such as heating rate and final temperature. It was reported that higher areas are obtained when heating rates below 1 °C/min and/or high gas space velocity (150,000 h^−1^) are used, as these conditions reduce sintering due to water release [13,14]. Other nitrogen sources such as urea or *m*-phenylenediamine have been employed in order to improve the efficiency of the nitridation [15,16].

The use of nitrogen-rich transition metal nitrides in catalysis is very promising. However, the synthesis of TMN with high nitrogen to metal ratio is energetically demanding. This has prompted researchers to find aternative methodologies and new structures such as polymorphs of Zr_3_N_4_, Hf_3_N_4_, Ta_2_N_3_, and noble metal dinitrides OsN_2_, IrN_2_, and PtN_2_ have been explored [17].

Sautet et al. [18] recently studied the nitridation of transition metal surfaces and tried to offer some insight into the synthesis and stability of transition metal nitrides under working conditions. The authors studied fifteen transition metals and using theoretical and experimental techniques evaluated the extent of nitridation (surface vs. bulk) depending on the metal and the shape of nanoparticles. By using the nitrogen chemical potential at which metal covered by nitrogen is more stable than bare metal, the authors were able to stablish a nitridation trend among the studied serie. As depicted in Figure 1, Mo, W, Fe and Re are more easily nitrided, becomign more difficult when moving to the right hand side of the periodic table, i.e., less oxophilic metals, which agrees well with previous experimental results [19,20].

**Figure 2:**
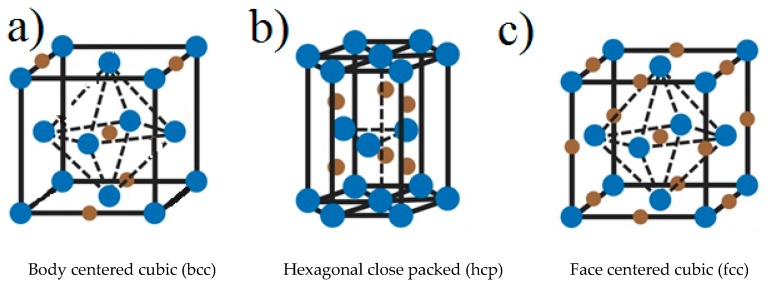
(**a**) bcc: TiN, ZrN, HfN, VN, CrN; (**b**) hcp: Mo_2_N, W_2_N; (**c**) fcc: MoN, TaN. Blue points represent transition metal atoms and brown points nitrogen atoms. Adapted from [6], with permission from Wiley, 2013.

The most studied transition metal nitride in catalysis is molybdenum. In general cubic γ-MoN_x_ (0.5 ≤ x < 1) is obtained by NH_3_ treatment of the oxide precursor, MoO_3_, while mixtures of H_2_ + N_2_ lead to the formation of tetragonal β-MoNx (x ≤ 0.5) or γ-MoNx. Hexagonal δ-MoNx (x ≥ 1) is obtained from MoS_2_ and NH_3_. Similarly, the content of nitrogen on iron nitrides influences their structure, so upon increasing the nitrogen content, the lattice structure changes from fcc γ’-Fe_4_N to hcp ε-FexN (2 < x ≤ 3) and to orthorhombic ζ-Fe_2_N. In Figure 2, the most common structures of transition metal nitrides are shown.

## 3. Transition Metal Nitrides as Catalyst

### 3.1. Hydrotreatment Reactions

Transition metal nitrides are considered excellent candidates to replace noble metals in hydrogen-treatment reactions since they show similar or even better performance than noble metals. It has been reported that Mo nitrides can easily chemisorb hydrogen due to the contraction of the d-band and the changes in the electron density that result as a consequence of the interstitial incorporation of N in the Mo metal lattice. Moreover, in the Mo_2_N-based catalysts hydrogenation reaction occurs over nitrogen vacancies, so Mo/N ratio also influences the catalytic performance [21]. 

Both CO and CO_2_ hydrogenations are very interesting reactions since they overcome key environmental challenges while providing energy or valuable chemicals. On the one hand CO hydrogenation can be employed to purify H_2_ gas streams before feeding to fuel cells to avoid poisoning. On the other hand, CO_2_ hydrogenation has been appointed as a solution to reduce the amount of CO_2_ evolved to the atmosphere from the industrial activity and convert it to fuels and chemicals [22,23]. 

However CO and CO_2_ hydrogenation face several challenges. CO_2_ activation is difficult due to the inert nature of the molecule and the cleavage of the C−O bonds in CO_2_ demands high activation energy. Also, methanation of CO and CO_2_ which provides an efficient alternative to conventional natural gas, is highly exotermic. The produced reaction heat favours metal sintering, which decreases the catalyst activity. Catalyst deactivation can also take place when carbon deposits on the active phase. 

Zaman et al. [24,25] have studied the influence of adding alkali promoters to the Mo_2_N systems in CO hydrogenation. The synthesis in both cases was performed by a simple temperature programmed treatment of the molybdenum and alkali precursors under ammonia flow. This leads to a material containing a mix of different phases: Mo, Mo_2_N, Mo oxide and alkali-Mo oxide phase.

The promotion with alkalis favoured the conversion to oxygenates, i.e., methanol, ethanol and propanol. Cesium was found to be better promoter to oxygenates at 5% wt. (28% selectivity to oxygenates vs. 11% with Li promoted and 6.5% with unpromoted). The lower selectivity achieved with Li compared to Cs was attributed to the formation of Li_2_MoO_4_ phases during nitridation. 

**Figure 3:**
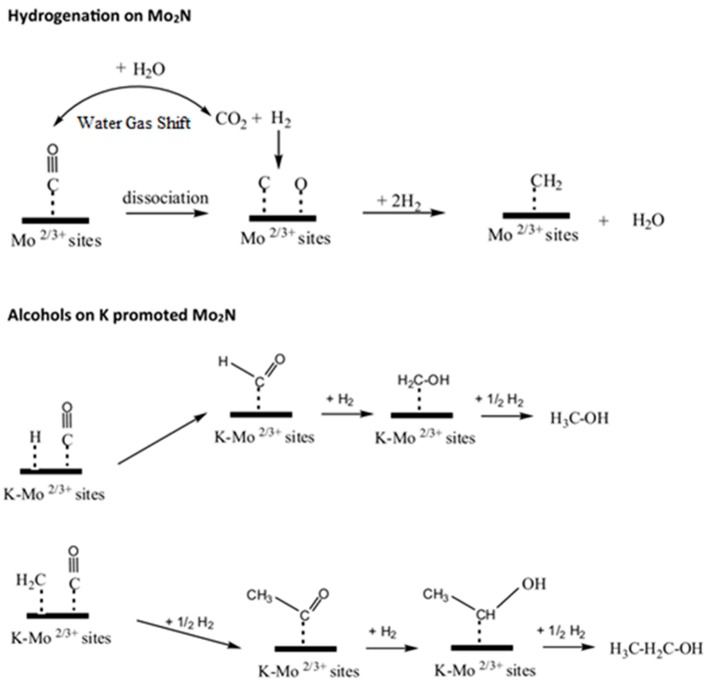
Plausible CO hydrogenation reaction pathways on Mo_2_N and K-Mo_2_N catalysts. Reproduced from [25] with permission from Elsevier, 2018.

On the other hand, bare Mo_2_N leads to the preferential conversion of CO to hydrocarbons, which can be ascribed to (i) CO dissociative hydrogenation and (ii) water-gas shift reaction, as shown in Figure 3. However, the presence of alkali hinders CO dissociation, which benefits the molecular insertion of CO into—CHx intermediate and promotes the coupling of alcohols.

Regarding the effect of alkali loading, the authors performed a thorough study with potassium as promoter using several weight percentages: 0.45, 1.3, 3 and 6.2% [24]. The best selectivity to oxygenates, 44%, was obtained over promoted K-Mo_2_N with a K/Mo surface ratio of 0.06 which also corresponded to the best K distribution among the samples. The XRD showed that both γ-Mo_2_N cubic and monoclinic K_2_MoO_4_ were formed and this latter phase seems to increase upon K addition further than 3% wt, this being detrimental for K distribution and hence for oxygenates conversion. 

Methanation, is the catalytic hydrogenation of carbon oxides (CO and CO_2_) to obtain synthetic natural gas. Ru, Rh and Ni catalysts have proven to offer good catalytic performance in terms of activity and selectivity to methane which can be further improved by using bimetallic systems and optimizing catalyst synthesis method [26]. 

Methanation and hydrodesulfurization of dibenzothiophene was studied over molybdenum nitride by Zhao et al. [27]. The authors were able to synthetize a rich nitrogen molybdenum nitride using high pressure, 3.5 GPa, through a solid-state ion exchange reaction. This new nitride, 3R−MoN_2_ holds a rhombohedral R3m structure, isotypic with MoS_2_. However it offered catalytic activities three times higher than MoS_2_ for the hydrodesulfurization of dibenzothiophene and over twice as high in the sour methanation of syngas at 723 K. 

The binary nitride, Ni_2_Mo_3_N, was studied by Leybo et al. [28] in the methanation of CO_2_. Yet, modest selectivites to CH_4_, ca. 20%, were obtained and the active phase suffered sintering upon reaction conditions, decreasing the stability of the catalyst. 

Besides molybdenum other metal nitrides have been tested in methanation reaction. For example co-methanation of CO and CO_2_ have been evaluated by Li et al. [29] over cobalt nitrides supported on alumina. The authors studied the effect of metal loading on Co_4_N/γ-Al_2_O_3_ and Co/γ-Al_2_O_3_ catalysts. According to the characterization, the cobalt nitride favoured stronger interactions with the support, this improving the dispersion of the nanoparticles and also their resistance to coking and metal sintering after 250 h which, as previously mentioned, is critical in such a exotermic reaction. Moreover, it was confirmed that the nitrogen atoms improved the adsorption of reactants due to their basicity, leading to better catalytic performance compared to the Co metal supported catalysts. The better results were also explained by the uniform metal dispersion and superior metal-support interaction. 

Fisher-Tropsh is a well-known transformation to convert syngas, CO + H_2_, into liquid hydrocarbons and that was very relevant in catalytic research in the 70–80s due to the oil crisis. Now, it has revised attention since it can also use syngas from biomass to produce fuels and chemicals. The most studied systems are those based on iron as they offer optimal results under a variety of conditions that allow to tune the selectivity, and are economically interesting. However, under reactions conditions the water produced can oxidize the catalyst with its subsequent deactivation [30].

**Figure 4:**
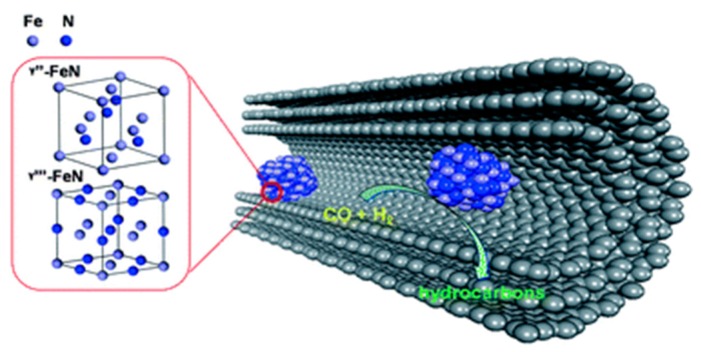
Reproduced from [32], with permission from Royal Society of Chemistry, 2011.

Bao et al. [31] studied the confination effect of FeN cubic nanoparticles inside carbon nanotubes (FexN-in) (see Figure 4). Firstly, FeN supported on CNT resulted 5–7 times more active than FeN supported on silica and metallic Fe on CNT. This seems to be related to the better stability of the nitrides under CO hydrogenation conditions compared to metallic and carbide iron which are oxidized by water, resulting in catalyst deactivation. Also, the catalyst where FeN nanoparticles were selectively loaded inside the CNT was more active than the catalyst with FeN nanoparticles mainly dipersed on the external walls, FexN-out, (1.4 times). The authors explained the better activity of the confined nanoparticles by the lower particle size and the formation of more FeCxN1x entities on FexN-in than on FexN-out during reaction, leading to stronger retention of nitrogen atoms in the lattice. 

In contrast to the activity and stability enhancement of iron nitride compared to its parent metal, cobalt nitride seems to be a poison for FT synthesis. In literature it was reported that by adding a nitrogen source such as acetonitrile or ammonia to the FT gas feed, cobalt nitride phases are formed which result in catalyst deactivation by deposition on the most active metallic cobalt sites (steps and edges) [32]. 

So far, theoretical results have predicted that CO adsorption and dissociation over γ-Mo_2_N(111) has a similar activation barrier to that of MoS_2_, so similar activity for syngas conversion can be expected [33].

CO_2_ dry reforming of methane (DRM) has received much attention, as it transforms two greenhouse gases (CH_4_ and CO_2_) into syngas. The most studied catalysts have been noble metals, Ru, Rh and non-noble metals such as Ni [34]. Some reports appeared using transition metal carbides due to their low cost and similar structure compared to noble metals. However at atmospheric pressure, carbides can easily be deactivated due to oxidation by CO_2_ or H_2_O [35]. Hence, alternatively nitrides have been tested as potential catalysts for DRM. 

Gu et al. [36] studied Mo_2_N, Ni_3_Mo_3_N and Co_3_Mo_3_N above 550 °C and atmospheric pressures and found that a synergic effect is observed in the bimetallic nitrides that improve the activity and resistance to oxidation and coke deposition on the DRM compared to the monometallic nitride Mo_2_N. The most active and stable catalyst was Co_3_Mo_3_N which, among other factors, was ascribed to the synergistic effect between the Mo and interstitial metal Co. 

Owing to environmental concerns and the depletion of fossil resources, in the last decade researchers have focus on the study of biomass derived compounds to obtain fuels and chemicals. Since the starting lignocellulosic biomass owns a high oxygen concentration (>50%), most of the transformations require selective removal of oxygen. More specifically, one of the most studied reactions is hydrodeoxygenation (HDO). Under HDO condititions, the reactants can be also converted through the decarboxylation/decarbonylation (DCO) path, promoting the C−C cleaveage which is undesirable for fuels and chemicals. 

For example, Monnier et al. [37] studied the conversion of oleic acid and canola oil with nitrides of Mo, W, and V supported on γ-Al_2_O_3_. The Mo_2_N catalyst exhibited superior activity for oleic acid conversion compared to the other nitride catalysts, and also favored the HDO route vs. DCO. The HDO path produces preferentially n-C_18_H_38_ (diesel fuel cetane enhancers). Also, Mo_2_N/γ-Al_2_O_3_ was stable in continuous hydrotreatment of canola oil at 400 °C under 83 bar hydrogen, reaching a constant yield of 50% middle distillates. 

Murzin et al. [38] studied Ni and Mo_2_N-MoO_2_ on the HDO of more complex reactants, *Chlorella* algal oil extracted with supercritical hexane and stearic acid, at 300 °C under 30 bar in the presence of hydrogen. The catalysts were selective to fatty acids, indicating deactivation of decarbonylation sites. The catalyst Mo_2_N-MoO_2_, despite being less active than the Ni based catalysts, was more stable and it showed no deactivation after a 360 min test. These results open new possibilities that should be explored regarding mixtures of nitrides and oxides. Nitrides are known to be deactivated in the presence of water due to oxidation, but these oxides might tolerate better the presence of impurities, enhancing their stability.

Zhang et al. [39] prepared cobalt nitride supported on a nitrogen doped carbon CoNx@NC using cellulose and ammonia as the carbon and nitrogen source respectively at different synthesis temperatures from 500 to 800 °C. The catalysts were tested in the HDO of eugenol at 2 MPa H_2_ and 200 °C. According to the reaction results, the reaction follows different paths when using nitrides or metallic cobalt. While the nitrides favour the cleaveage of the C-aryl−OCH_3_ bond to form 4-propylphenol, metallic cobalt promotes the hydrogenation of the alkene moiety. The best catalyst was CoNx@NC-650 which displayed the largest surface area and dispersion of the nitrides nanoparticles. The catalyst was also successfully tested to promote the HDO of phenolic compounds.

**Figure 5:**
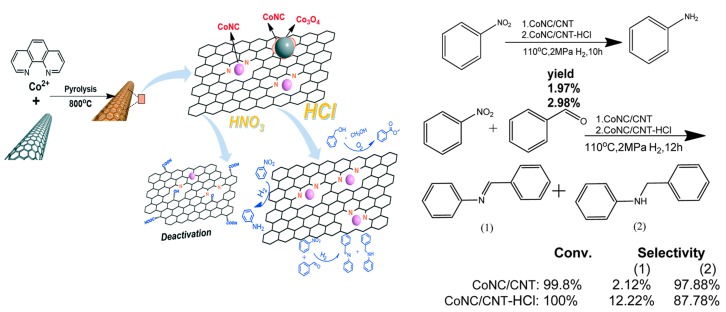
CoNC/CNT active sites on nitro compounds hydrogenation and hydrogenated coupling of nitrobenzene with benzaldehyde. Reproduced from [41], with permission from Royal Society of Chemistry, 2016.

Supported CoNx on carbon nanotubes on the hydrogenation of nitrobenzene and hydrogenated coupling of nitrobenzene with benzaldehyde was studied by Zhang et al. [40] as schematized in Figure 5. Some catalytic tests verified that catalytic activity was mainly due to the CoNx entities and the authors also suggested that the activity was mainly due to the cobalt chelate complexes bonded to nitrogen atoms of the graphene lattice. 

Lodeng et al. [41] compared the activity of molybdenum nitride, carbide, and phosphide supported on TiO_2_ on the HDO of phenol at 25 bar and in a temperature range between 350 and 450 °C. All the catalysts were highly active to benzene and only minor amounts of aromatic ring hydrogenation were obtained. Molybdenum nitride displayed lower activity compared to its carbide and phosphide counterparts, but its selectivity to cyclohexene was higher than that of phosphide and similar to carbide. 

Hydrogenation of −COH moieties constitutes one of the most interesting and studied reactions in fine chemistry since it allows obtaining a high number of compounds that are used for example in pharmaceuticals and/or fragances. Reactants such as cinnamaldehyde or crotonaldehyde have been widely studied in an attempt to heterogeneized the catalytic system. The catalysts must be selective to the unsaturated alcohols without reducing the C=C bonds. With that aim heterogeneous catalysts based on noble metals mainly Ru, Pd and Pt have been widely investigated with good results in terms of activity and selectivity [42] and the significant role of nitrogen improving the selectivity to the desired products have also been reported [43]. To date, these specific transformations has been tested with bimetallic systems of metal nitrides and noble metals. 

**Figure 6:**
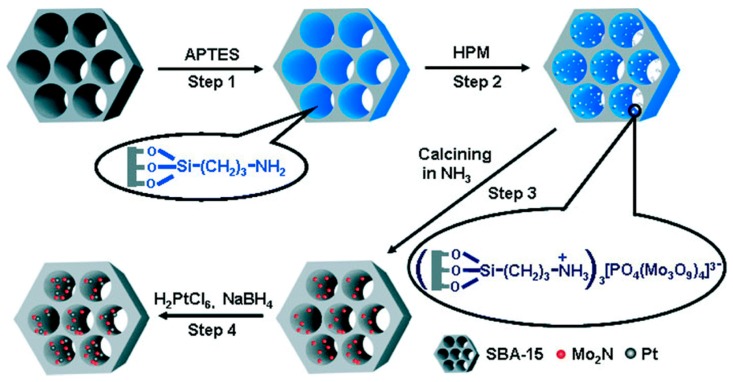
Synthesis of Pt-Mo_2_N-SAB-15. Reproduced from [45], with permission from American Chemical Society, 2016.

Fu et al. [44] have used a previously functionalized support to obtain small nitrides nanoparticles supported on the mesoporous silica SBA-15. The synthesis of Mo_2_N over SBA-15 started by functionalizing the support with a monoamine that is located homogeneously into the pores of the support as shown ion Figure 6. This amine is then used as anchoring point for the molybdenum precursors which preferentially adsorbs on the moieties. Then, the procedure follows the previously explained temperature reduction procedure in NH_3_. Finally the noble metal is impregnated and reduced with NaBH_4_ forming bimetallic phases with Mo_2_N. In this way, Mo_2_N and Pt nanoparticles with a size of about 8.0 and 5-6 nm respectively were obtained with metal loadings of Mo (22% wt.) and Pt (3% wt.). 

Then, Pt/Mo_2_N/SBA-15 with different Pt loadings (1–3% wt.) was tested in the chemoselective hydrogenation of cinnamaldehyde. Both activity and selectivity to the cinnamyl alcohol was higher over Pt/Mo_2_N-based catalysts than over monometallic Pt/SBA-15, that the authors ascribed to the synergy between Pt and Mo_2_N nanoparticles and the more efficient use of the Pt surface on the bimetallic sample. 

In the work of Thomson et al. [45], some more insight was given regarding the reaction mechanism and nature of active phases. To do so, the authors used a thorough experiment by hydrogenating a high surface area γ-Mo_2_N to obtain a partially hydrogenated entity γ-Mo_2_N-Hx. Then, by combining H_2_-TPD experiments and DFT simulations three different hydrogen species were identified: surface nitrogen bound (κ1-NHsurf), surface Mo bound (κ1-MoHsurf) and subsurface Mo-bound (μ6-Mo6Hsub). 

**Figure 7:**
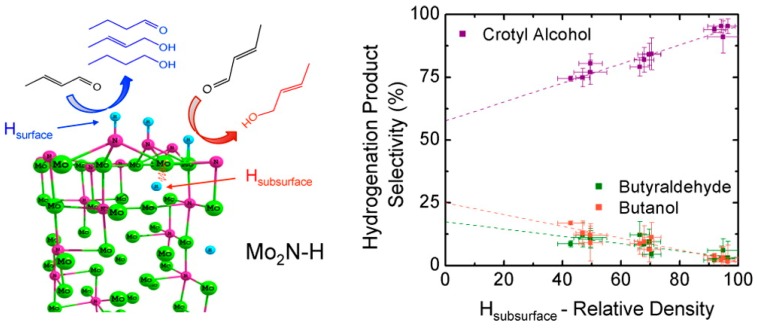
Reproduced from [45], with permission from American Chemical Society, 2016.

The reactivity of these species was assessed by testing them in the hydrogenation of crotonaldehyde. Accordingly, the authors proposed that reaction starts by a heterolytic dissociation of H_2_ to form surface NH (κ1-NHsurf) and MoH (κ1-MoHsurf) as schematized in Figure 7. Then, since subsurface interstitial H site (μ6-MoHsub) is more energetically favored than surface κ1-MoHsurf, hydrogen migrates into the lattice. Moreover, based on the catalytic results the authors proposed that surface and subsurface species MoH (κ1-MoHsurf/μ6-MoHsub) are more selective to the hydrogenation of the C=O bond and the surface κ1-NHsurf sites hydrogenate preferentially the C=C bond. 

The selective hydrogenation of acetylene to ethylene is an important transformation since ethylene is the monomer to produce polyethylene polymers and it has a strategic relevance in refineries, being critical its high purity production. 

The catalytic behaviour of β- and γ-Mo_2_N in the partial hydrogenation of acetylene was evaluated by Lizana et al. [46] that studied the influence of synthesis parameters on textural properties of the nitrides and its effect on the catalytic performance. The results showed that selectivity of both β- and γ-Mo_2_N was higher than over Pd-based catalysts. Also, β-Mo_2_N which displays higher surface Mo/N ratio compared to γ-Mo_2_N, offered lower selectivity to partial hydrogenation and a two-fold higher specific acetylene hydrogenation rate. 

Altarawneh et al. [47], used computational methods to study mechanism of the selective hydrogenation C_2_H_2_ over γ-Mo_2_N to C_2_H_4_ rather than complete hydrogenation to the corresponding alkane.

**Figure 8:**
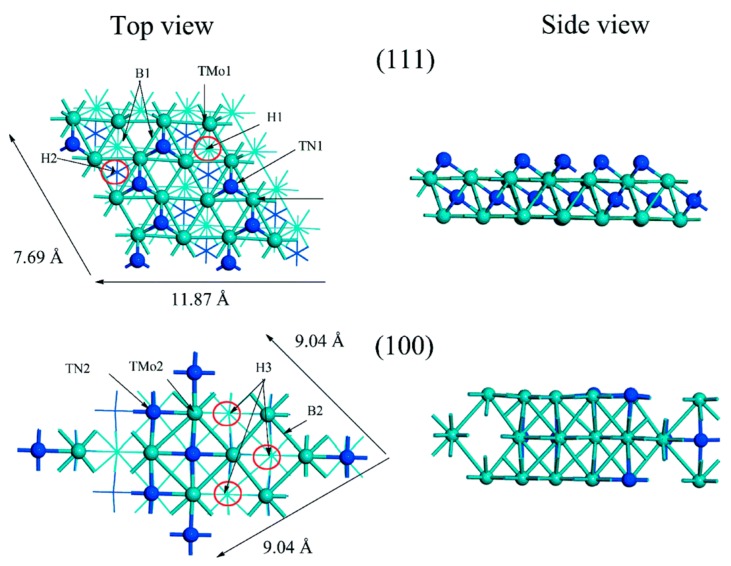
H_2_ adsorption sites on Mo_2_N (111) and (100) faces. Reproduced from [48], with permission from American Chemical Society, 2016.

Reactions take place through H_2_ adsorption followed by dissociation. The authors obtained the modes of H_2_ adsorption as shown in Figure 8: 3-fold hollow fcc (H1) and 4-fold hollow fcc (H3) sites over the (111) and (100) terminations of γ-Mo_2_N, respectively. 

In agreement with experimental results, this work seems to confirm that dissociation of H_2_ occurs over nitrogen vacancies. It is also proposed that the lower stability of the partial hydrogenated molecule, C_2_H_4_ leads the selectivity.

Another interesting reaction within fine chemistry is the hydrogenation of nitroaromatic compounds, since aromatic haloamines are important intermediates in the manufacturing of drugs, pesticides, and pigments among others. The reaction has been successfully performed over Au [48], Ir [49] and Pd [50] catalysts supported over a variety of materials.

Keane et al. [51] demonstrated experimentally that Mo_2_N improved the performance of Au in the selective hydrogenation of *p*-chloronitrobenzene (p-CNB) to *p*-chloroaniline (p-CAN) reaching 100% selectivity to p-CAN, a four-fold higher hydrogenation rate compared to Au/Al_2_O_3_ and showed stability upon several cycles. 

**Figure 9:**
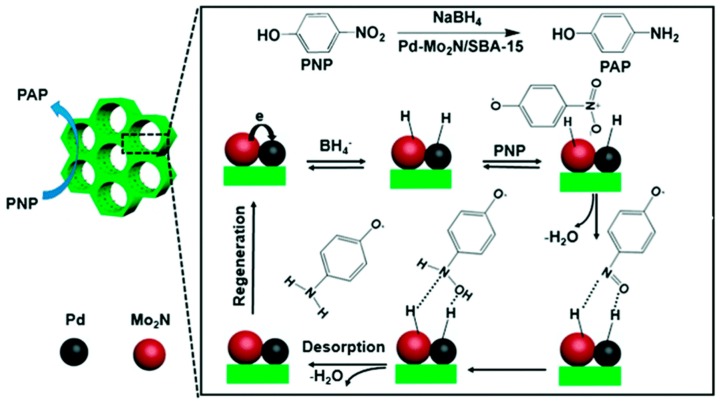
Catalytic conversion mechanism of PNP into PAP over the Pd–Mo_2_N/SBA-15 hybrids in the presence of NaBH_4_. Reproduced from [53], with permission from American Chemical Society, 2018.

The system Pd/Mo_2_N was an effective catalyst for the hydrogenation of p-nitrophenol (PNP) to p-aminophenol (PAP) [52]. In this study, the authors synthetized Pd/Mo_2_N nanoparticles of 2–3 nm size over SBA-15. The high dispersion improved the interaction between Pd and the nitride so that 1 wt% Pd–Mo2N/SBA-15 showed better catalytic performance than 1 wt% Pd/SBA-15 and 20 wt% Pd/SBA-15. In Figure 9 the proposed reaction mechanism over the Pd–Mo_2_N/SBA-15 is shown.

Wu et al. [53] reported a green solvent-free synthesis method for CoNx entities supported on doped mesoporous carbon materials (CoNx-OMC) with surface areas in the range 678–1250 m^2^/g, high N content (4.3–10.8 wt%) and rich in CoNx sites as verified by XPS. The optimized CoNx-OMC, thermally treated at 800 °C) catalyst showed an interesting catalytic performance on the hydrogenation of several nitro compounds, i.e., 100% conversion, almost 100% selectivity and stable upon recycling, under mild conditions (5 bar H_2_ pressure, 110 °C). According to the catalytic and characterization results, a synergy effect is reached between CoNx sites and the nitrogen doped support. On the one hand, the CoNx entity provides specific sites for the adsorption and activation of nitro groups. On the other hand, the nitrogen heteroatoms of the support act as anchoring sites, increasing the dispersion of the active components and facilitating mass transportation. Also, it was confirmed that cobalt nitride was responsible of the activity and not the metallic Co nanoparticles.

Density functional theory calculations were performed by Altarawneh et al. [54] who studied the hydrogenation of p-CNB to p-CAN over the model γ-Mo_2_N(111) surface. The results showed that adsorption of p-CNB is thermodynamically favoured over Mo-hollow face-centered cubic (fcc) and N-hollow hexagonal close-packed (hcp) sites with adsorption energies of −32.1 and −38.5 kcal/mol, respectively. Also, the results are in agreement with previous experimental reports that described a high selectivity to p-CAN at low temperatures, the direct path being preferential versus the condensation route [55]. 

The theoretical results suggest that activated hydrogen, H*, is transferred from both fcc and hcp hollow sites to the NO/−NH groups, the hydrogenation of chloronitrosobenzene being the rate-limiting step with an energetic barrier of 55.8 kcal/mol. Also, the high energy barrier for direct fission of the C−Cl bond excludes the formation of aniline.

Another industrial transformation of acetylene is the hydrochlorination to obtain vinyl chloride monomer (VCM), the basic unit of polyvinyl chloride (PVC). Industrially this reaction is performed using HgCl_2_ as catalyst, however due to environmental and health concerns, alternative systems have been evaluated [56]. Among them, noble metal, i.e., Au, Pd, Pt and Ru over activated carbon, have been the most studied, gold being the best in terms of activity and selectivity. 

Since it has been previously found that nitrogen doping can promote adsorption for HCl [57], which is the VCM synthesis reaction rate-controlling step, a potential catalytic system is that consisting of metal nitrides. 

Dai et al. [58] studied V, Mo, and W nitrides (10 wt% metal loading) supported on activated carbon. Their experiments showed that VN/AC offered very low activity in the reaction. Mo_2_N was initially the most active, but deactivated with time to give lower conversion values than those reached with W_2_N/AC. The selectivity to C_2_H_3_Cl was similar for all the tested nitrides and reached near 100%. To further explain these results, TPD of the reactants, C_2_H_2_ and HCl, was performed and showed that W_2_N/AC dislayed a stronger and similar interaction with both reactants, compared to the other catalysts. However, Mo_2_N/AC which deactivates during reaction, showed an easy desorption of HCl and difficult desorption of C_2_H_2_, this producing significant amounts of coke which can be responsible of the observed deactivation. 

The authors also studied binary Mo and Ti nitrides with different Mo/Ti ratios supported on activated carbon for acetylene hydrochlorination [59]. All the binary nitrides displayed better catalytic performance compared to the mononitrides and a Mo/Ti ratio of 3 was optimal among the studied systems offering 89% conversion and selectivity over 98.5%. Apparently a synergy effect among Mo and Ti occurs so that adsorption of HCl is enhanced while adsorption of acetylene is reduced. 

Other chloro compounds have been evaluated with TMN. For example Keane et al. [60] studied the gas phase hydrodechlorination of 1,3-dichlorobenzene (1,3-DCB) using molybdenum and tungsten carbide (Mo_2_C, W_2_C) and nitride (Mo_2_N). fcc-Mo_2_N showed better activity (by a factor of 20) compared to pure hcp- and fcc-carbides, that displayed similar activity. 

### 3.2. Oxidation

The selective oxidation of carbon monoxide with low concentration of O_2_ (CO-PROX) is used to purify hydrogen rich streams obtained from hydrocarbon reforming. So far, the most studied and active system are the CuO–CeO_2_ and Pt based catalyst [61]. 

With the aim of finding more economical active and selective catalysts, the catalytic performance of several transition metal nitrides have been assessed. For example, Yang et al. [62] studied the effect of Co loading (1 to 10 wt%) on Co_4_N supported on γ-Al_2_O_3_. The cobalt nitrides displayed similar activities compared Pt-group metals in the temperature range 200–220 °C. The sample 3 wt% Co/γ-Al_2_O_3_ offered the best activity and selectivity in PROX reaction which could be related to the higher concentration nitrogen vacancies in the near-surface that enhance the adsorption of reactants. 

Selective oxidation of alcohols plays an important role in many industrial transformations such as energy conversion and storage or the production of fine chemicals. The most studied and active catalysts are supported Au Pt, Pd, Ag and Ru and other non-noble-metal catalysts such as Co and Cu [63]. 

In order to test more economically viable alternatives Deng et al. [64] evaluated the catalytic activity of iron nitride, FeN_4_, supported on graphene in the oxidation of benzene using H_2_O_2_ as oxidant. The characterization suggested that atomically dispersed FeN_4_ were obtained and these entities were also stable after reaction. The catalyst reached 23.4% conversion and 18.7% yield of phenol at room temperature, however no comparison with other catalyst is given.

Later, Yuan et al. [65] studied several metal nitrides (MNx/C-T, M = Fe, Co, Cu, Cr, and Ni) synthetized at different pyrolysis temperatures, T, in the oxidation of unsaturated alcohols. The most active and selective catalyst to the corresponding aldehydes was the iron nitride. Among them, the catalysts prepared by thermal treatment at 900 °C displayed the better catalytic performance in the selective oxidation of HMF to DFF with almost complete conversion and selectivity exceeding 97%. According to the characterization performed the authors suggested that when thermal treatment was performed at lower temperatures, c.a. 600 °C, a higher concentration of nitrogen doped carbon was formed, in detriment of iron nitride; and that nitrogen doped carbon offered lower activity. In contrast, the formation of FeN_4_ was favored at higher synthesis temperatures, resulting in materials that offered better activity. Moreover, the activity of the recycled catalysts could be restored by thermal treatment under NH_3_/N_2_. 

With the aim of gaining more insight into the active sites of the Fe−N−C catalysts. Zhang et al. [9] studied atomically dispersed Fe−N−C catalyst synthetized using nano-MgO as a template. The catalysts were tested in the selective oxidation of the C−H bond of a wide range of aromatics, heterocyclic, and aliphatic alkanes at room temperature. The catalysts showed high activity and selectivity of up to 99%, as well as great reusability. The atomical dispersion of FeNx (x = 4 − 6) was verified using sub-Ångström-resolution HAADF-STEM along with XPS, XAS, ESR, and Mössbauer spectroscopy.

**Figure 10:**
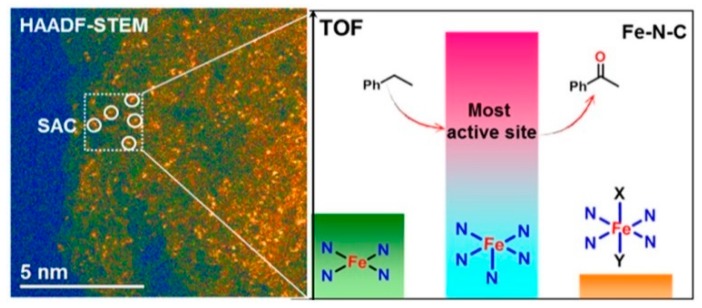
Reproduced from [9], with permission from American Chemical Society, 2017.

The authors also reported that the concentration of each FeNx species depends on the pyrolysis temperature. Among the studied samples, the most active was Fe−N−C-700 which is comprised of high-spin FeN_6_ (28.3%), low-spin FeN_6_ (53.8%), and medium-spin FeN_5_ (17.9%) species, as shown in Figure 10. This latter is over one order of magnitude more active than the other two species. Upon increasing the pyrolysis temperature, the concentration of FeN_5_ decreases to less than 10%, leading to lower activity. 

### 3.3. Ammonia Synthesis and Decomposition

The production of ammonia through the Haber–Bosch Process was a technological breakthough of the last century since it is a relatively easy way of obtaining a synthetic fertiliser. The process uses promoted iron catalyst to produce ammonia from N_2_ and H_2_ at temperatures of ca 400 °C and high pressure of around 100–200 atmospheres. The process of ammonia synthesis is so relevant that it currently consumes near 1–2% of the world’s energy demand [66]. 

This data reveals that more efforts can be made to optimize the process as small changes on the reaction conditions could have a huge impact on global energy consumption. Thus, despite being a well-known process, several attempts have been performed to obtain more active and stable catalysts to work under milder conditions [67]. 

In the last decades binary and ternary transition metal nitrides of the type Mo, Co and Fe have been tested in both ammonia decomposition and ammonia synthesis. Simulations and experimental tests have shown that cobalt molybdenum nitride, Co_3_Mo_3_N can be the most active catalyst for ammonia synthesis, superior to the industrial iron catalyst and promoted ruthenium catalyst [68,69]. 

The synthesis of ammonia catalyzed by molybdenum nitride seems to be a structure-sensitive reaction so that the bigger the particle size, the higher the intrinsic activity of the catalyst [70]. On the other hand, the effect of the nanoparticle morphology does not seem to be so clear. Sun et al. studied the synthesis of ammonia over plate-like γ-Mo_2_N and nanorod β-Mo_2_N and γ-Mo_2_N [71] and did not find significant differences among them. Also, the synthesis conditions limit the comparison since residual sulphur from the molybdenum precursor, Mo_2_S, can poison the catalyst.

In order to increase the dispersion of the nitrides and improve the stability, Ding et al. [72] synthetized molybdenum nitride supported on HZSM-5, using MoOx as precursor and NH_3_ as nitriding agent at 973 K. In this way molybdenum oxide exchanges with hydroxyl groups on the zeolite surface and obtained a Mo to N ratio close to 2. The authors proved that this supported molybdenum nitrides are more stable against oxidation by comparing with unsupported catalysts. 

The resulting catalyst, MoNx/ZSM-5, displayed excellent activity in the ammonia synthesis under ambient pressure. A kinetic approach showed that the apparent activation energy for ammonia synthesis on MoNx/ZSM-5 is around 20% lower than that on bulk Mo_2_N (9.8 kcal/mol vs. 12.4 kcal/mol).

Interestingly, the effect of reaction pressure was different on bulk Mo_2_N and MoNx/ZSM-5, i.e., higher reaction pressures are more favorable on the MoNx/ZSM-5 catalyst than on γ-Mo_2_N. The authors suggested that this difference could be due to the interaction of nitrogen with the zeolite framework, acting as an equivalent pressure which may enhance the pressure effect on the ammonia synthesis. Another significant finding of this work is that upon increasing the Si/Al ratio of the zeolite, the catalytic activity of MoNx increases. 

Recently, Catlow et al. [73] used DFT calculations to evaluate the associative and dissociative mechanisms of ammonia synthesis over Co_3_Mo_3_N. The results showed that in the associative mechanism, Eley-Rideal/Mars van Krevelen, hydrogen reacts with nitrogen adsorbed and activated on the surface to generate ammonia. 

Promotion of cobalt molybdenum nitrides with other elements have been also studied. According to Paweł Adamski [74] chromium and potassium were able to generate a well-developed porous structure, increasing the activity of the catalysts in ammonia synthesis by 50% compared to the non-promoted catalyst.

Ammonia decomposition has become a strategic research topic since it allows to obtain CO free hydrogen to feed fuel cells. Ammonia can also be employed directly as fuel in vehicles since it has a high energy density (8.9 kW h/kg), is easily liquified at room temperature and low pressure, i.e., less than 10 bar, and its narrow combustion range allows safe operation. 

Many cataytic monometallic and bimetallic systems have been tested based on Fe, Ni, Co, Ru, Ir, Pt or Rh, supported on several materials. Among them, ruthenium catalysts supported on different carbon materials such as active carbon, carbon nanotubes showed the highest activity.

In all cases, researchers observed a low activity for ammonia decomposition reaction at temperatures below 400 °C, since recombinative desorption rate of the adsorbed N atoms from active metals is slow at those conditions [75]. Also, hydrogen molecule seems to cover active sites over Ni- and Ru catalysts, this hindering the ammonia decomposition reaction. Hence, despite being well studied systems, the temperatures required and the use of noble metals should be avoided to make the process technical and economically feasible. 

Eguchi et al. [76], studied the effect of a second transition metal on Mo nitride based catalysts, prepared by temperature-programmed reaction under NH_3_ flow of the oxides precursors: MoO_3_, CoMoO_4_, NiMoO_4_, and FeMoO_4_. Incorporation of the second metal into the Mo nitride resulted in a significant decrease in the surface area (3.1–8.8 vs. 80 of Mo_2_N). However, the area of Mo_2_N was reduced to 23 m^2^/g after reaction, indicating that the material was not stable under the reaction conditions. Despite the lower surface area, the addition of a second metal was beneficial for ammonia decomposition and the activity followed the trend Co_3_Mo_3_N > Ni_3_Mo_3_N > Fe_3_Mo_3_N > Mo_2_N. Several transition metal nitrides have been studied for ammonia decomposition, being the binary system metal-cobalt molybdenum nitride the most interesting among them [77,78,79,80,81].

Based on the NH_3_-TPSR results, the authors suggested that the addition of Co and Fe favoured the desorption of hydrogen. However, over Mo_2_N de desorption of nitrogen was slower since nitrogen atoms tend to interact stronger with the nitride. According to the results the authors concluded that the order in which each metal nitride system dissociates metal nitride–N bond was Co_3_Mo_3_N ≈ Ni_3_Mo_3_N > Fe_3_Mo_3_N > Mo_2_N. Moreover, the presence of Co, Ni, and Fe improves the stability against poisoning by H_2_ on the active sites and the best results are obtained when doped catalysts are employed.

In order to improve the surface area of nitrides, alternative synthesis paths have been explored. For example Podila et al. [77] studied the use of citric acid as chelating agent to prepare bulk Co_3_Mo_3_N, with surface areas in the range 93–129 m^2^/g. The use of citric acid (CA) as chelating agent afforded better nitride dispersion which resulted in better catalytic performance. An optimal concentration of citric acid was related to a higher surface area, lower particle size and increased proportion of Mo_2_N and Co_3_Mo_3_N phases, so that when the CA/Mo ratio was changed from 1 to 3 in the synthesis, the conversion increased from 75% to 97% at 550 °C. 

Similarly Zaman et al. synthetized nickel [78] and cobalt molybdenum nitrides [79] using citric acid and compared its catalytic performance on ammonia decomposition with that obtained employing γ-Mo_2_N. Under these synthesis conditions, the surface area of the binary nitrides was increased but still below 20 m^2^/g. According to the results both catalyst, Ni_2_Mo_3_N and Co_3_Mo_3_N offered over 97% conversion, while the use of pure γ-Mo_2_N resulted in 50–70% conversion under the same experimental conditions. 

Zhao et al. [80] used supported binary CoMo nitrides over several porous materials with different physico-chemical characteristics: CNTs, Al_2_O_3_, activated carbon and 5A Zolite. The experimental procedure was a simple impregnation of the precursors followed by a temperature-programmed reaction in N_2_–H_2_. The activity followed the order: CoMoNx/CNTs > CoMoNx/Zeolite 5 > CoMoNx/AC > CoMoNx/Al2O3. However, despite the clear better performance of CNT, the supports differ in many features such as surface chemical composition and morphology, making the comparison and related conclusions quite difficult.

The effect of synthesis conditions, nitridation temperature and iron loading was also evaluated in iron nitrides supported on carbon nanotubes [81]. A higher synthesis temperature of ca. 500 °C and Fe loading of 10% prepared under NH_3_ flow resulted in well-dispersed Fe_2_N nanoparticles which exists along with Fe_2_O_3_ entities and offered the best ammonia conversion among the catalysts tested. In contrast the synthesis under a N_2_/H_2_ flow resulted in the formation of both Fe_2_N and Fe_4_N, this latter species being detrimental for ammonia decomposition. 

## 4. Conclusions

This short review of the most recent literature has shown that transition metal nitrides possess a wide spectrum of catalytic applications with interesting catalytic performance. The review has focused on thermal heterogeneous catalysis which requires high surface areas and good dispersion of the active phase. Despite the significant advances done up to date, there is plenty of room for research on transition metal nitrides to optimize the synthesis conditions in order to obtain higher surface areas and better nanoparticles dispersion, ideally reducing the synthesis temperature. 

Also, further work to improve the stability will be required in order to obtain potential industrial catalysts. One of the main reason for deactivation is the oxidation of the nitrides, which is likely to occur in reactions that generate water such as carbon dioxide hydrogenation, or when water is already in the reactants mixtures as it happens with biomass transformations. In this sense, it seems that subsurface hydrogen can delay deactivation and more insight into this reaction mechanism would allow to propose regeneration mechanism. 

Again, the use of a high surface area support for transition metal nitrides nanoparticles can improve the dispersion of the active phase and potentially improve their stability upon reaction conditions against sintering and oxidation. 

The improved catalytic performance that has been reached with more complex systems that incorporate a second or third metal, should be complemented with deeper understanding of the actual active phase and the chemical structure of the nitrides. Similarly, the use of promoters like alkalis and its effect of the structure need to be further studied since these materials have also demonstrated a significant potential for future catalytic applications. However, there is still no clear correlation mainly due to the complexity of these systems and difficulties to perform in situ investigations.
